# Variational autoencoder-based estimation of chronological age and changes in morphological features of teeth

**DOI:** 10.1038/s41598-023-27950-4

**Published:** 2023-01-13

**Authors:** Subin Joo, Won Jung, Seung Eel Oh

**Affiliations:** 1 Department of Medical Robotics, Korea Institute of Machinery and Materials, Daegu, 42994 South Korea; 2 Department of Oral Medicine, School of Dentistry, Institute of Oral Bioscience, Jeonbuk National University, Jeonju, 54896 South Korea; 3 Research Institute of Clinical Medicine of Jeonbuk National University - Biomedical Research Institute of Jeonbuk National University Hospital, Jeonju, 54907 South Korea; 4 Research Group of Consumer Safety, Korea Food Research Institute, Wanju, 55365 South Korea

**Keywords:** Biomedical engineering, Data processing, Image processing, Machine learning

## Abstract

This study led to the development of a variational autoencoder (VAE) for estimating the chronological age of subjects using feature values extracted from their teeth. Further, it determined how given teeth images affected the estimation accuracy. The developed VAE was trained with the first molar and canine tooth images, and a parallel VAE structure was further constructed to extract common features shared by the two types of teeth more effectively. The encoder of the VAE was combined with a regression model to estimate the age. To determine which parts of the tooth images were more or less important when estimating age, a method of visualizing the obtained regression coefficient using the decoder of the VAE was developed. The developed age estimation model was trained using data from 910 individuals aged 10–79. This model showed a median absolute error (MAE) of 6.99 years, demonstrating its ability to estimate age accurately. Furthermore, this method of visualizing the influence of particular parts of tooth images on the accuracy of age estimation using a decoder is expected to provide novel insights for future research on explainable artificial intelligence.

## Introduction

Morphological changes in teeth over time provide substantial evidence when estimating the age of unidentified individuals; thus, approaches using such data have been widely used in various applications, such as forensic dentistry and age estimation for immigrants^1^. Various methods based on forensic dentistry that use teeth for age estimation have been proposed. Statistical approaches, in which the morphological features of teeth are quantified and statistically analyzed, have been accepted as conventional^2,3^. This approach makes it possible to estimate age based on tooth data obtained from unidentified individuals with no age information. Based on this statistical data, the subjects’ age can be estimated. However, this method of quantifying the morphological features of subjects' teeth using auxiliary indices through manual work and estimating their age based on the obtained dimensional information generally takes quite a long time. It may lead to different outcomes depending on the proficiency of operators who analyze teeth radiographic images^4–6^.

To overcome these problems, various studies have been conducted to develop artificial intelligence (AI) models that can automatically extract the features of teeth via deep learning using orthopantomograms (OPGs) and estimate the age based on the obtained feature values^5,7–9^. Among various machine-learning-based methods for extracting feature values from medical images, a deep learning analytical method based on a convolutional neural network (CNN) has proved highly effective in diagnosing subjects’ symptoms based on medical images or estimating the age of bones^10^. These AI models, developed based on OPG and CNN, are less affected by their users' proficiency and can be used to estimate the age of subjects based on a large amount of data in a relatively short time^11,12^. However, CNN-based algorithms lack interpretability because users often find it challenging to determine which part of a given tooth image has affected the age estimation result or the causal relationship between aging and morphological changes in teeth^1,5,13^.

Vila-Blanco et al.^5^ and Kim et al.^1^ developed CNN algorithms capable of estimating the age of subjects using OPGs. They further employed a Gradient-weighted Class Activation Mapping (Grad-CAM) technique to identify the regions of teeth that significantly affect the results of age estimation models. When estimating the age of teeth, the Grad-CAM technique indicates the degree of importance of each part of the input dental radiographs as a heatmap, thus allowing users to easily spot the parts of the given images of all or individual teeth that are important for age estimation. Milošević et al.^13^ developed age prediction models based on the existing CNN structure, which had already proven highly effective. This study developed various prediction models, such as individual tooth-based models and models with removed teeth, to determine which regions of the given OPG images contained useful age indicators. Further, each model's age estimation accuracy was compared and analyzed. However, these methods have several limitations. They still fail to visualize the correlation between human aging and the morphological changes in teeth with high-resolution images, and the correlation between the quantitative properties of age and morphological properties of teeth lacks clear interpretability. Zhang et al.^14^ and Zhao et al.^15^ developed an autoencoder-based model capable of both age estimation and result interpretation to enhance the interpretability of deep learning algorithms. Further, it verified the performance of the developed model using face and brain images. The present study investigated the use of an autoencoder to provide clearer interpretability of the effects of human aging on teeth.

This study proposes a method for extracting feature values from teeth images using AI algorithms and by estimating the subjects' age based on the extracted feature values. It also determines which regions of the teeth are more important than others for age prediction based on dental radiographs. A variational autoencoder (VAE), specially designed to extract feature values from images, was used to develop feature-value-based prediction models and methods for data interpretation. The VAE was trained using the first molar and canine tooth images obtained from previous studies. In addition, a method for quantitatively determining and analyzing the correlation between age and morphological changes in the teeth was proposed. Specifically, the age estimated by the VAE was compared with the amount of morphological variation between consecutive teeth images over time, that is, with advancing age.

## Experimental methods and procedures

### Dataset

OPG data obtained from 910 Korean outpatients treated at the Jeonbuk National University Dental Hospital were used as research data. Informed consent was obtained from all participants, and all methods were performed in accordance with the relevant guidelines and regulations. This study was approved by the Institutional Review Board of the Jeonbuk National University Hospital (CUH 2021-03-021). The age of the participants was determined based on the date of radiography. The age ranges of the patients are presented in Table 1. Based on a previous study, in which mandibular first molar and canine teeth were used for age estimation, in the present study, each image was cropped into four sub-images of two types of teeth on the left and right^1,13,16^. More specifically, the images were manually segmented under the guidance of skilled dentists into four sub-images of left and right mandibular first molar teeth and left and right mandibular canine teeth. The obtained images were then resized to 256 × 256 pixels to obtain maximum pixel information. From a total of 910 dental radiographs, 1216 images of first molar teeth and 1634 images of canine teeth were obtained. Herein, when these tooth images were used to train the developed model, all the left-side images were mirrored before use to ensure that the learning process was not affected by whether a given tooth was located on the left or right side^17,18^.

**Table 1:** Age and gender distribution of dental x-ray datasets.

Age group	Gender		Total
	Male	Female	
10–19	30	54	84
20–29	79	116	195
30–39	23	66	89
40–49	57	97	154
50–59	53	111	164
60–69	74	73	147
70–79	33	44	77
Total	349	561	910

### Variational autoencoder-linear regression model

#### Model overview

This study developed a model for estimating the age of a subject based on a dental radiograph dataset, and visually pinpointed the specific region of the teeth that influences the estimation. A model that combined a VAE and linear regression was used to provide the region with clearer interpretability compared to the use of the convolutional neural network (CNN) and Grad-CAM techniques. The VAE consisted of an encoder and a decoder. The encoder was used to extract image features with unique latent variables, and the decoder made it possible to regenerate the original images using these latent variables. Linear regression could be used to develop a model that could estimate the age of a subject using the latent variables obtained from the encoder. In addition, after adding the regression coefficient to each latent variable, the decoder could use these latent variables to generate an image of the region of interest in the teeth. This study referred to the combined VAE and linear regression model as the “VAE with linear regression,” which could be further classified into either “Single VAE with linear regression” or “Parallel VAE with linear regression,” based on how the VAE was structured.

#### Single VAE with linear regression

Convolutional neural networks (CNNs) are powerful deep learning tools that can automatically extract feature values from input image data to establish a nonlinear relationship model between label data and the corresponding feature values^19^. A convolutional VAE is a probabilistic graphical model capable of extracting features of input image data as a continuous probability distribution function using two symmetrical CNNs^20^. When the dental radiograph $x$ is entered into ${q}_{\varnothing }(z|x)$, which is the VAE encoder model, the probability distribution of all latent z variables, which correspond to all characteristic values that the input data can result in, is then returned as the output. The decoder ${p}_{\theta }(x|z)$, also referred to as the generative model, returns virtual image data $\overline{x }$, reconstructed from the input latent z variables.

To train this VAE model, the Kullback–Leibler divergence that minimizes the distribution of latent z variables between $q(z|x)$ and $p(z)$ was first calculated, as shown in Eq. (1). Next, the reconstruction error is calculated, as shown in Eq. (2), to minimize the difference between $x$, the dental radiograph that was entered into the encoder, and $\overline{x }$, the resultant image obtained from the decoder. The total loss of the VAE model ${\mathcal{L}}_{VAE}$ is used to train the model with hyperparameters in a manner that minimizes both the KL divergence loss ${\mathcal{L}}_{KL}$ and image reconstruction loss ${\mathcal{L}}_{rec}$, as shown in Eq. (3). To optimize the learning process of the developed model, each loss term was weighted by the factor γ.

1 $${\mathcal{L}}_{KL}= {D}_{KL}(q(z|x)||p(z))$$

2 $${\mathcal{L}}_{rec}= -{\mathbb{E}}_{q\left(z|x\right)}[\mathrm{log}p(x|z)]$$

3 $${\mathcal{L}}_{VAE}= {{\gamma }_{1}\mathcal{L}}_{KL}+ {\gamma }_{2}{\mathcal{L}}_{rec}$$

**Figure 1:**
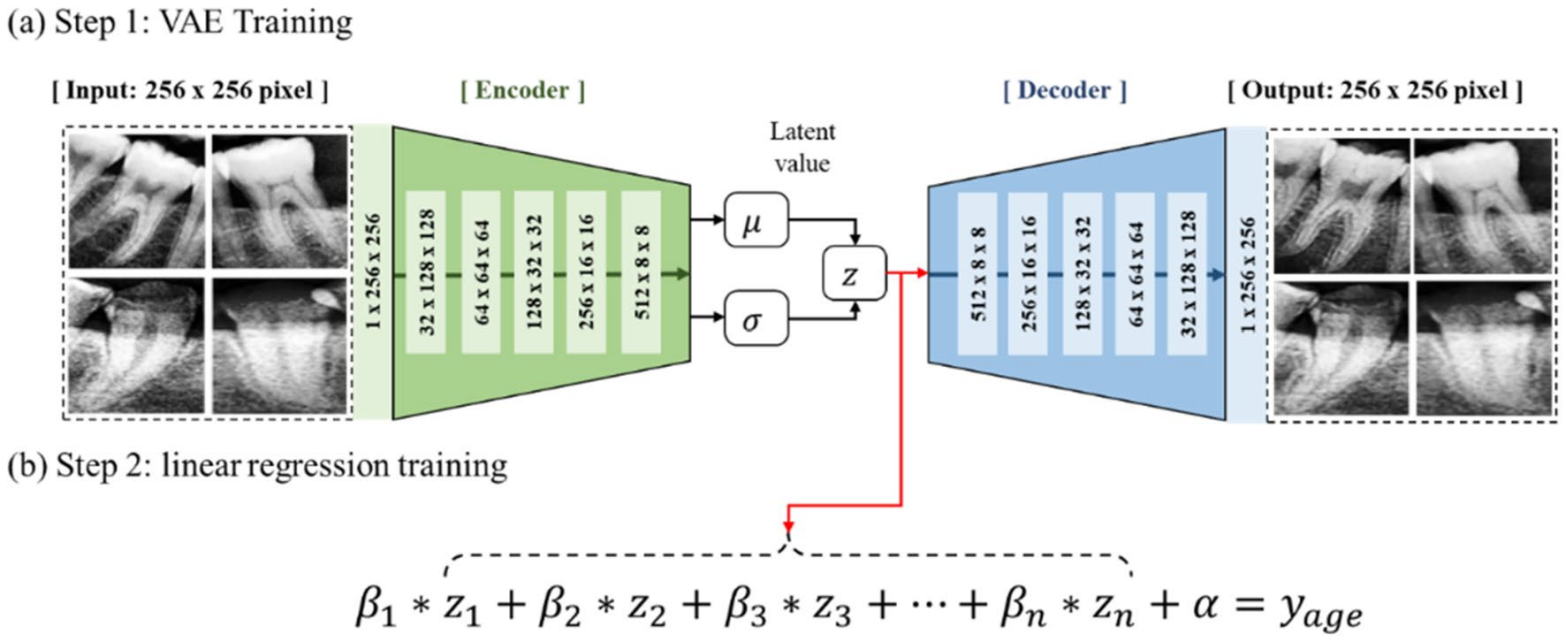
Structure of and training method for the variational autoencoder and linear regression model. (**a**) VAE model that can extract latent z variables from tooth images. (**b**) Regression model that can estimate age from latent z variables.

In this study, a VAE composed of five convolutional layers was developed, and grayscale images of 256 × 256 pixels were used as the input for the model, as shown in Fig. 1a. From the input images, the encoder model returned 512 × 8 × 8 distribution variables $\mu$ and $\sigma$. The latent z variables were then calculated from the obtained distribution variables. The decoder model receives the latent z variables as input and generates virtual images of 256 × 256 pixels as the output. A linear regression equation was developed to estimate the age of the teeth using the latent z variables obtained from the encoder, as shown in Fig. 1b. The VAE model was built using Python's TensorFlow Library and trained in an unsupervised learning manner. The linear regression equation was developed using Python's Scikit-learn Library and trained in a supervised manner.

#### Parallel VAE with linear regression

Accuracy and precision are among the most critical factors determining the performance of dental age estimation methods in adults, especially from the perspective of forensic odontology. The accuracy of these methods may vary depending on the tooth selected and examine or the estimation method employed. Thus, selecting and applying at least two estimation methods is important to improve accuracy and precision^21^. This is also the case for dental age estimation using AI techniques; multiple teeth must be comprehensively examined. Therefore, the present study developed a parallel VAE model capable of estimating the age of subjects by comprehensively analyzing the feature values obtained from at least two types of tooth images, generating a virtual image of the teeth^22,23^.

**Figure 2:**
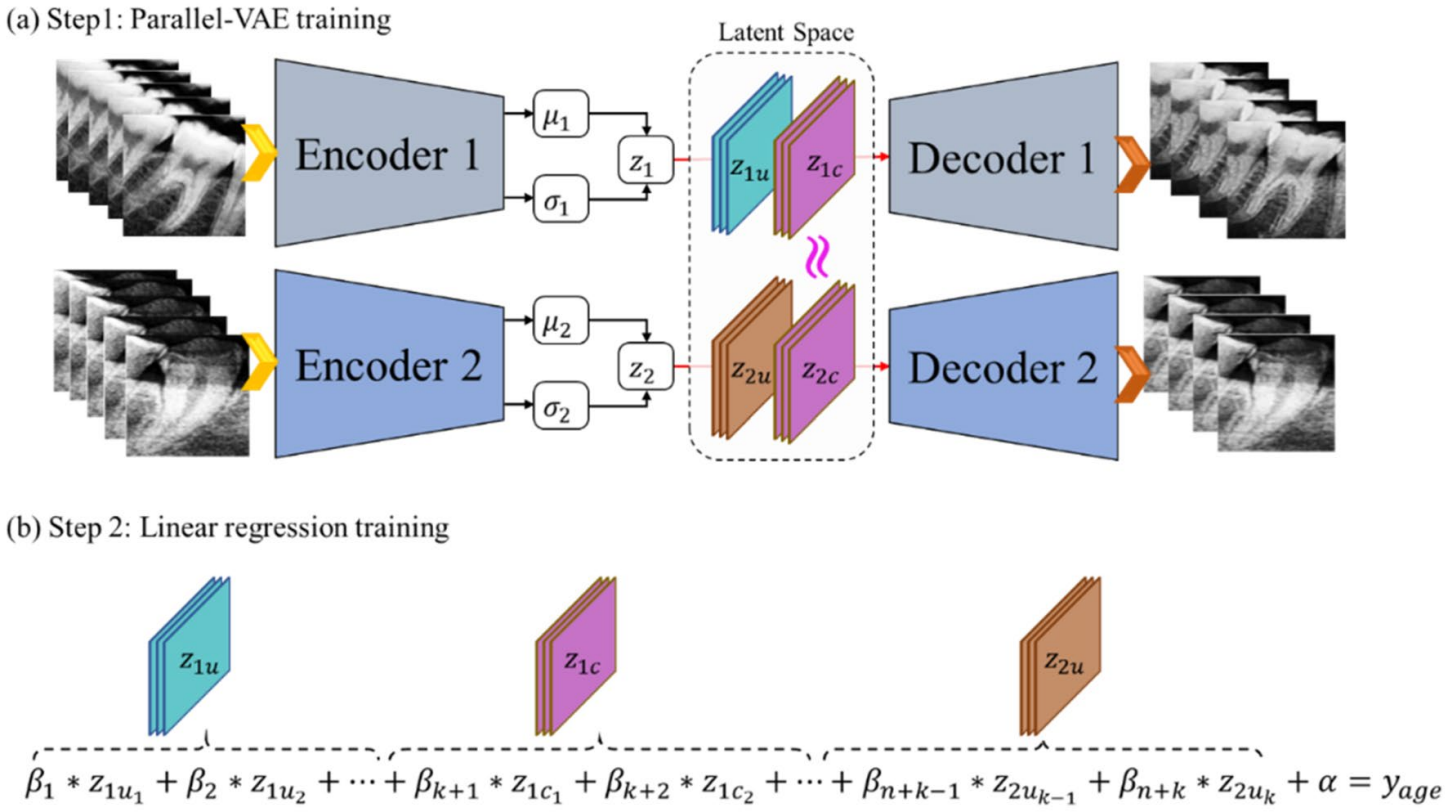
Structure of and training method for the parallel-variational autoencoder and linear regression model. (**a**) Parallel-VAE model that can extract common latent variables and unique latent variables from two tooth images. (**b**) Regression model that can estimate age from two unique latent variables and one common latent variables.

Assuming that images of the first molar and canine obtained from a single subject contained common latent variables that may help estimate the subject's age, a parallel VAE model that partly shared the same latent z variables was developed, as shown in Fig. 2a. Encoder 1 and Decoder 1 of the parallel VAE used first molar images as input and output data, respectively. Encoder 2 and Decoder 2 used datasets of canine images as input and output data, respectively. Here, it was assumed that, among the $\mathcal{n}$ latent variables calculated from Encoder 1, ${z}_{1u}$, which corresponded to half the $\mathcal{n}$ latent variables ($\mathcal{k})$, included unique variables specific to first molar teeth, and the other half of the latent variables (${z}_{1c}$) included common variables shared by both first molar and canine teeth. Similarly, among the $\mathcal{n}$ latent variables obtained from encoder 2, ${z}_{2u}$, which corresponded to half the $\mathcal{n}$ latent variables ($\mathcal{k})$, were assumed to include unique variables specific to canine teeth, and the other half (${z}_{2c}$) was assumed to have common variables shared by both the first molar and canine teeth. To ensure that the latent variables ${z}_{1u}$ and ${z}_{2u}$ would have respective feature values for each tooth type while allowing the latent variables ${z}_{1c}$ and ${z}_{2c}$ to include common information shared by both types of teeth, Eq. (4) was added to the VAE loss calculation term^22^.

${\mathcal{L}}_{separate}$, which is the separate loss, was obtained by dividing the difference between the common variables ${\mathcal{L}}_{common}$ for the two tooth types by the difference between the unique variables ${\mathcal{L}}_{unique}$. The mean square errors between two latent variable sets can be expressed as in Eqs. (5) and (6). ${\mathcal{L}}_{{KL}_{m}}$ and ${\mathcal{L}}_{{rec}_{m}}$ in Eq. (7) refers to the KL loss and image reconstruction loss of the VAE that receives the first molar images as input. ${\mathcal{L}}_{{KL}_{c}}$ and ${\mathcal{L}}_{{rec}_{c}}$ are the KL loss and image reconstruction loss of the VAE that receives the canine as the input. If the entire parallel-VAE model is trained using the loss functions in Eq. (7) and the gradient descent methods, the common latent variables for each tooth type, that is, ${z}_{1c}$ and ${z}_{2c}$, tend to converge toward the same value gradually to minimize the ${\mathcal{L}}_{common}$ value. However, at the same time, this learning process proceeds in a way that maximizes the ${\mathcal{L}}_{unique}$ value, allowing the values of the unique latent variables for each tooth type, that is, ${z}_{1u}$ and ${z}_{2u}$, to be as different as possible.

4 $${\mathcal{L}}_{separate}= \frac{{\mathcal{L}}_{common}}{{\mathcal{L}}_{unique}}$$

5 $${\mathcal{L}}_{common}=\frac{1}{\mathcalligra{k}}\sum_{i=1}^{k}{({z}_{{1c}_{i}}-{z}_{{2c}_{i}})}^{2}$$

6 $${\mathcal{L}}_{unique}=\frac{1}{\mathcalligra{k}}\sum_{i=1}^{k}{({z}_{{1u}_{i}}-{z}_{{2u}_{i}})}^{2}$$

7 $${\mathcal{L}}_{parallel-VAE}= {{\gamma }_{1}\mathcal{L}}_{K{L}_{m}}+ {{\gamma }_{2}\mathcal{L}}_{K{L}_{c}}+{{{{\gamma }_{3}\mathcal{L}}_{re{c}_{m}}+\gamma }_{4}\mathcal{L}}_{re{c}_{c}}+{{\gamma }_{5}\mathcal{L}}_{separate}$$

A linear regression model capable of age estimation using the latent variables obtained from parallel VAE was developed, as illustrated in Fig. 2b. The configuration of this linear regression model was basically the same as that employed for the single VAE in that it received latent variables as input and returned the age of the subjects as output. However, this regression model was built using only 3/4 of the latent variables. This configurational difference is attributed to the fact that once the parallel VAE is sufficiently trained, the common latent variables ${z}_{1c}$ and ${z}_{2c}$ become almost identical; thus, there is no need to use both to develop a regression model. Ultimately, the developed regression model contained a total of $\mathcalligra{n}+\mathcalligra{k}$ regression coefficients $\beta$, along with $\alpha$, a single intercept.

### Generation of teeth images that reflect age changes

This study led to the development of a method for quantitatively determining and analyzing the correlation between age and morphological changes in teeth by generating dental images that vary continuously with age. This was accomplished using the coefficients of a regression model trained with age data and a decoder. Once fully trained, the regression model can be expressed as in Eq. (8).

8 $${\beta }_{1}*{z}_{1}+{\beta }_{2}*{z}_{2}+{\beta }_{3}*{z}_{3}+\dots +{\beta }_{n}*{z}_{n}+\alpha ={y}_{age}$$

**Figure 3:**
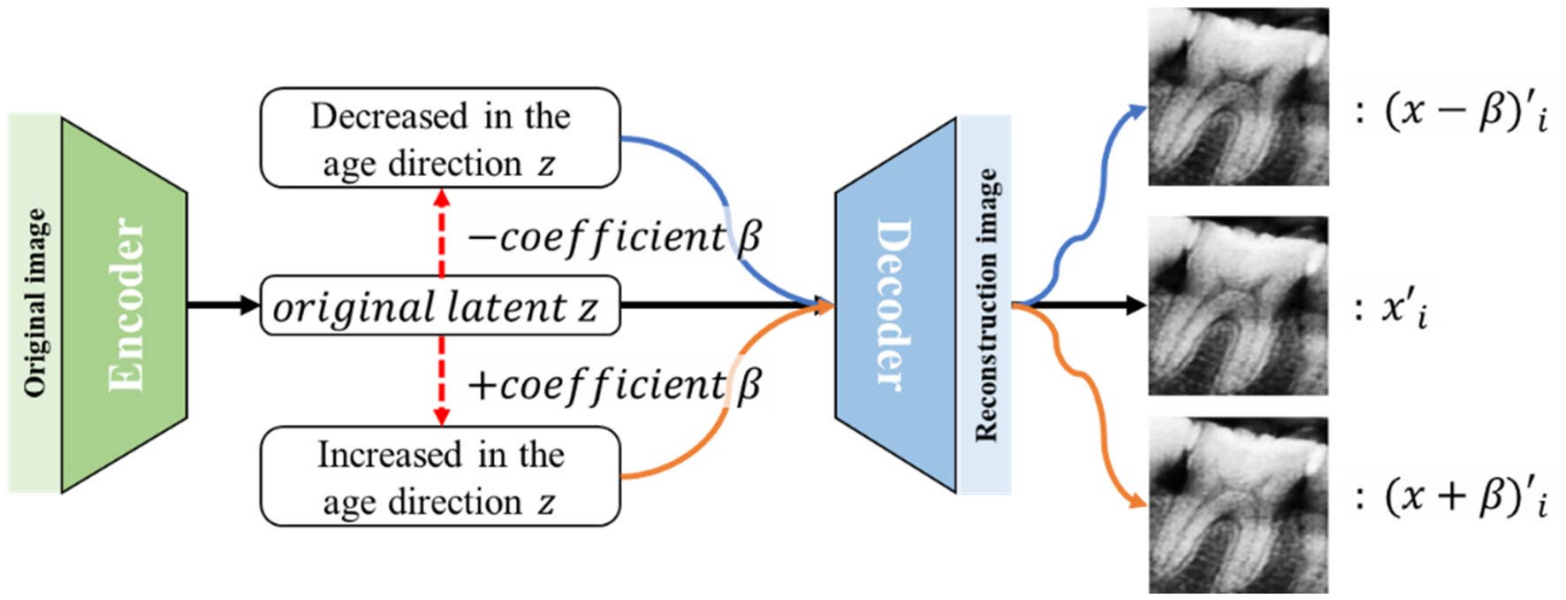
A method for visualizing changes in the morphology of teeth with age using the coefficients obtained from the trained linear regression model. β, the coefficient of the regression model trained with the latent variables and actual age data, represents the correlation between the latent variables and age. Thus, when adding or subtracting the coefficient β to or from the original latent variables generated from the reference image, the values of the latent variables that have a strong correlation with age tend to change greatly, while the values of those that have a weak correlation with age change less significantly. Therefore, the values of the latent z variable change according to the degree of correlation with age, and this relationship can be visually demonstrated using the decoder.

The regression coefficient ${\beta }_{k}$ may be used to estimate age and to generate latent variables corresponding to teeth images when the subject is younger or older than the reference image. The latent variables extracted from the encoder contain various types of information, including the brightness of the teeth images used, sex, and age. When a regression model is developed using age as a dependent value, the coefficients of latent variables that strongly correlate with age tend to be highly positive or negative. When the correlation with age is weak, the coefficients tend to be small. Based on this relationship between the latent variables and their coefficients, it is possible to selectively control only the values of the latent variables that strongly correlate with age. For example, when adding or subtracting the regression coefficient $\beta$ to or from the latent variables obtained from the reference image, the values of the latent variables that had a strong correlation with age tended to change significantly (Fig. 3). When the correlation is weak, the corresponding changes are small. Accordingly, virtual images in which the subject is younger than the reference image can be generated by subtracting the regression coefficient $\beta$ from the extracted latent variables and then reconstructing them using a decoder. Similarly, virtual images in which the subject is older can be obtained by adding the regression coefficient $\beta$ to the latent variables and reconstructing them using a decoder.

## Result

### Determining weights for the parallel VAE loss

**Figure 4:**
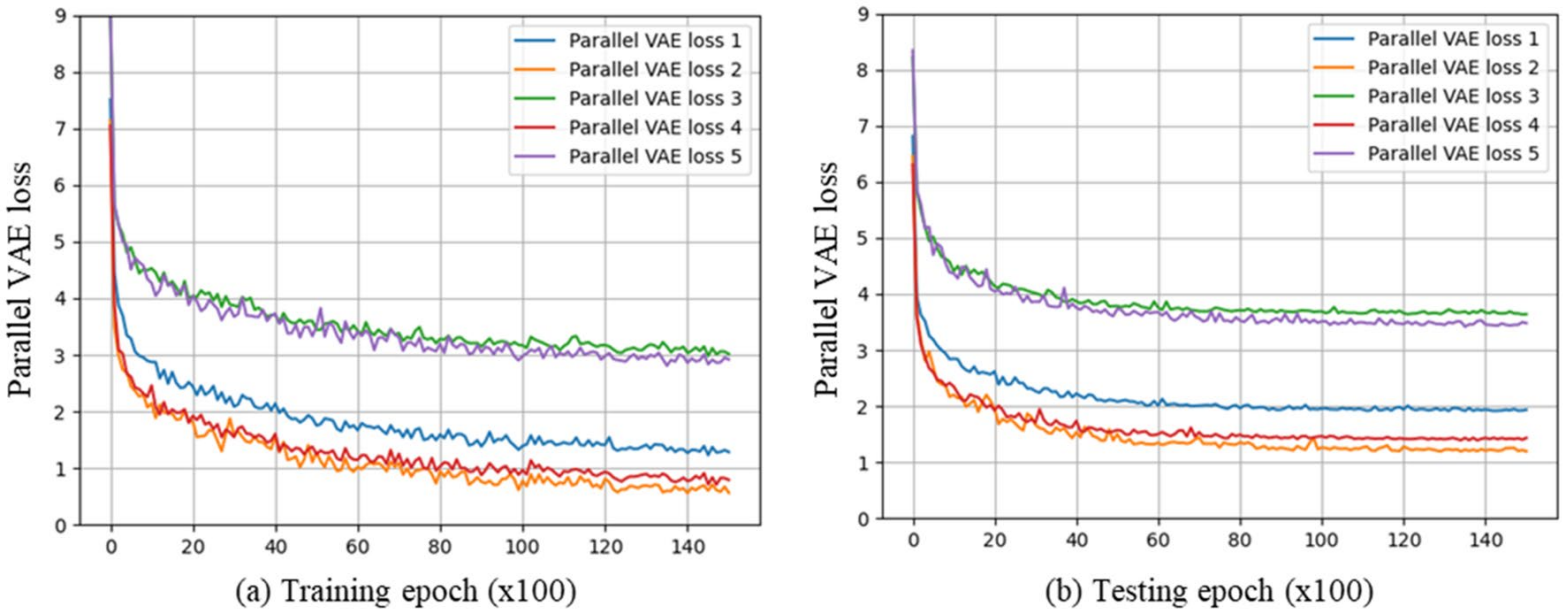
Results of the five combinations of weight values on parallel VAE loss values: (**a**) effects on parallel VAE loss during training and (**b**) effects of inputting test images to the model on parallel VAE loss values during training.

In this study, the weight values for the image reconstruction loss and KL divergence loss were set at 1 and 0.00001, respectively, to train a single VAE. As listed in Table 2, a total of five different combinations of weights were generated by tuning each of the weight values for the image reconstruction loss and KL divergence loss using scales of 10 and 1/10. Parallel VAE loss 1 was the case where the same weight values used to train a single VAE were used for ${\gamma }_{1}$, ${\gamma }_{3}$ (training on the first molar data), and ${\gamma }_{2}$, ${\gamma }_{4}$ (training on the canine data). From parallel VAE loss 2 to parallel VAE loss 5, the weight values for training the first molar and canine data were tuned using a scale of 10 to study the effects of different weight values on the learning process. The results of training with these five different combinations of weight values are shown in Fig. 4. Parallel VAE loss 2 had the highest age prediction accuracy out of all the prediction models trained with different combinations. Thus, it can be observed that setting a smaller weight for the first molar data in the parallel VAE training resulted in a better performance. Based on a comparison of the effects of the number of training epochs on the loss values for parallel VAE loss 2 and parallel VAE loss 4, it could be inferred that the image reconstruction loss and KL divergence loss obtained from the first molar data were smaller than those obtained from the canine data. Therefore, because the loss values obtained from the canine data were greater, setting smaller weights for ${\gamma }_{1}\mathrm{ and }{\gamma }_{3}$ (training on the first molar data) resulted in a better performance for the model by increasing the relative effects of the canine training data on the parallel VAE loss function.

**Table 2:** Five combinations of weight values for training with the first molar and canine to study the effects on parallel VAE loss values.

Cases	Weight of the VAE part learned using the first molar		Weight of VAE part learned using canine		Age prediction accuracy
	${\gamma }_{1}$	${\gamma }_{3}$	${\gamma }_{2}$	${\gamma }_{4}$	Mean
*Parallel VAE loss 1*	*0.00001*	*1*	*0.00001*	*1*	*8.69*
Parallel VAE loss 2	0.000001	0.1	0.00001	1	**8.24**
Parallel VAE loss 3	0.001	10	0.00001	1	8.31
Parallel VAE loss 4	0.00001	1	0.000001	0.1	8.48
Parallel VAE loss 5	0.00001	1	0.001	10	8.82

Significant values are in bold and italics.

### Age estimation

In this study, two models, single-VAE and parallel-VAE, were developed to determine more effective age estimation methods, especially when multiple types of teeth were comprehensively considered. The single-VAE model was designed to be trained using a combined dataset of first molar and canine tooth images. Thus, the latent variables of this model were trained to consider the feature values of both tooth types. By contrast, the parallel-VAE model was designed to be trained with unique latent variables, which reflect the respective feature values of each type of tooth, and common latent variables, which reflect the feature values shared by both types of teeth. The age of the subjects was estimated using the VAE as follows: Using an encoder that had been fully trained, latent variables were extracted from the given tooth images, and the extracted values were then entered into the regression model to estimate the age.

All the test subjects in the present study were divided according to age into seven groups with 10-year intervals. The age estimation accuracy was analyzed by age group, as shown in Table 3. Overall, the parallel VAE model was more accurate than the single-VAE model for age estimation. The median absolute error (MAE) was the lowest, at 5.32, for the thirties age group. The estimation error of both models decreased as the age range increased from teens to thirties but increased in the age groups. This result was consistent with the results of a study by Milošević et al.^13^ that analyzed the accuracy of age estimation based on single-tooth images by age group.

**Table 3:** Prediction accuracy of each training model, MAE is the median absolute error.

Age range	Parallel-VAE			Single VAE		
	MAE	Mean	std	MAE	Mean	std
10–19	10.02	9.90	5.67	12.53	13.62	7.36
20–29	6.73	8.02	5.83	10.38	11.07	7.20
30–39	5.32	5.96	3.80	5.53	6.72	5.07
40–49	5.64	6.07	4.81	5.95	7.18	5.64
50–59	7.25	7.94	5.46	8.07	9.37	6.46
60–69	10.43	11.48	7.89	14.21	14.07	8.18
70–79	12.84	13.31	8.27	22.31	20.51	7.50
All	6.99	8.24	6.10	9.50	10.62	7.49

Here, the estimation error was measured to be 10 years or more for subjects in their sixties or above. This result was also consistent with that of a previous study. For children, the accuracy of age estimation based on eruption stages is relatively high; however, the estimation is more difficult for adults because all their permanent teeth have fully erupted. Thus, the error in age estimation was generally higher for adult subjects^18^. In addition, adults’ eating habits and living environments increasingly affect their teeth cumulatively over time. Thus, the older the subjects, the larger the error and discrepancy in age estimation become^24^.

Table 4 lists the results of a comparison of the age estimation performances of the proposed parallel VAE with the linear regression model and methods from previous studies using identical datasets. Using the pre-trained VGG16 model, models were considered that utilized the first molar and canine data for age estimation. In addition, the results of another model were compared to those of a study that used a combined dataset of the two types of teeth images. The fact that the model developed with the first molar data showed better performance than the model developed with the canine data made it possible to infer that first molar data captured more significant properties of teeth with closer relationships to human aging^13^. In addition, the classification accuracy of the proposed method was compared with that of a previous study that classified subjects into either three groups or five groups, based on their ages, using the ResNet-152 model and voting method. The lower overall results obtained in the comparative experiments in this study compared to the results presented in the original papers could be attributed to a relatively higher distribution of teeth images from older demographics, which are generally more challenging to use for accurate age estimation^26^. As a result, it could be observed that using the parallel VAE structure for the age estimation model led to a better performance than those obtained by the methods proposed in previous studies. This outperformance may have been achieved because the parallel VAE structure was more effective at disentangling the specified feature values from other feature values^22,27,28^.

**Table 4:** Results of experiment to compare the performances of proposed methods, including regression and classification methods.

	Tooth type	Mean absolute error (years)		Classification accuracy (%)	
		Mean	std	3 Groups	5 Groups
Regression					
Proposed method	First molar & canine	**8.24**	**6.10**	–	–
VGG16 (Milošević et al. 2022)	First molar & canine	9.76	7.17	–	–
	First molar	8.40	6.44	–	–
	Canine	12.36	8.17	–	–
Classification					
Proposed method	First molar & canine	–	–	**80.28**	**74.33**
ResNet-152 (Kim et al. 2021)	First molar & canine	–	–	78.06	69.38

Significant values are in bold.

### Analysis of generated images

**Figure 5:**
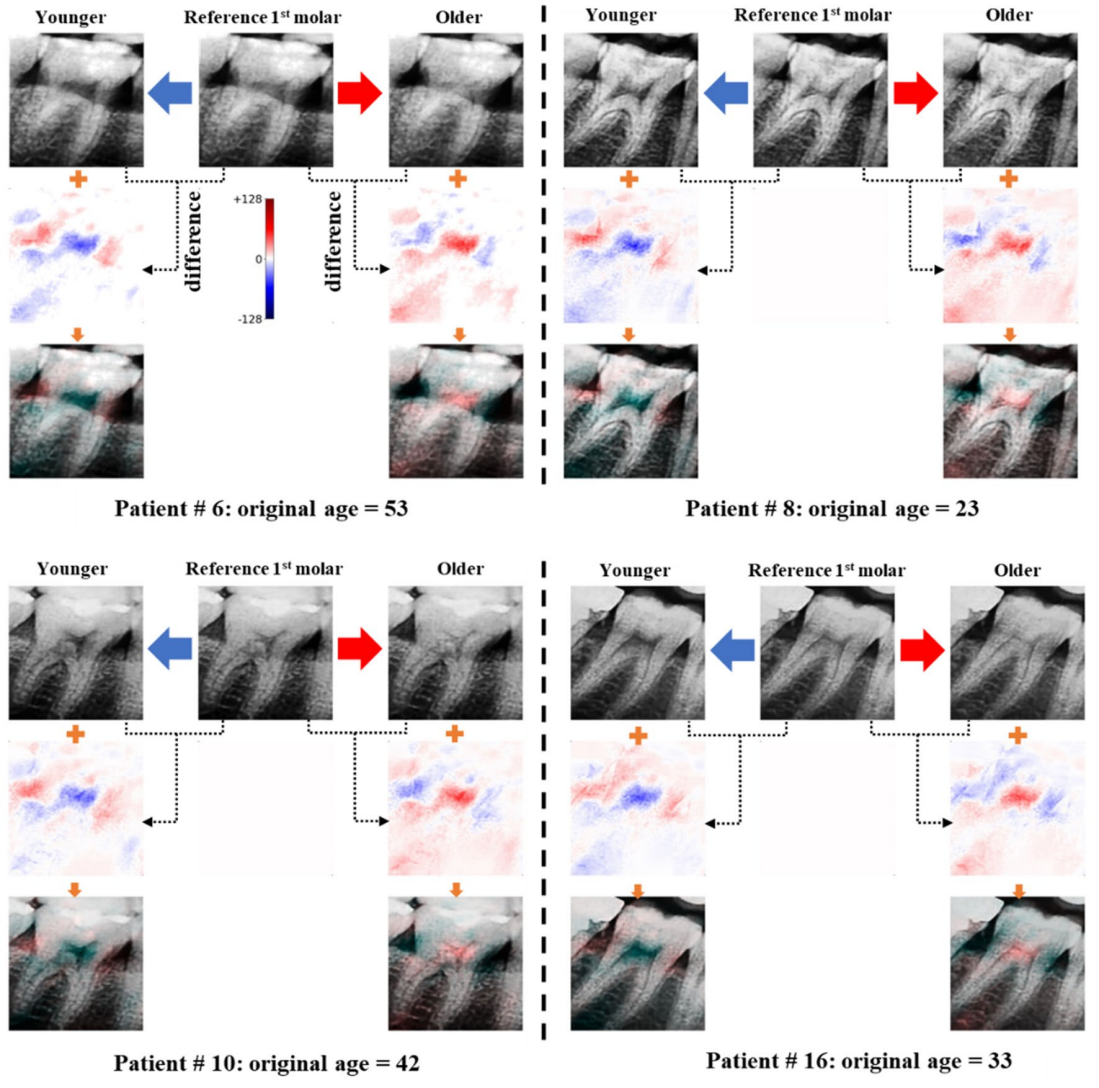
An example of the color maps that represent the correlation between advancing age and the morphological changes in teeth, prepared by generating virtual teeth images when the subject was younger or older using the developed VAE and linear regression model. The images in the first row are virtual images generated by controlling the latent variables of first molar teeth. The images in the second row are images in which the pixel difference between the original image and the virtual image generated by increasing or decreasing the age is expressed as different colors in the form of a diverging color map. Here, colors with higher intensity indicate that the corresponding regions have a stronger correlation with age. The images in the third row are those generated by overlaying the obtained color maps onto the corresponding teeth images to allow a quantitative analysis of the morphological changes in teeth with age.

**Figure 6:**
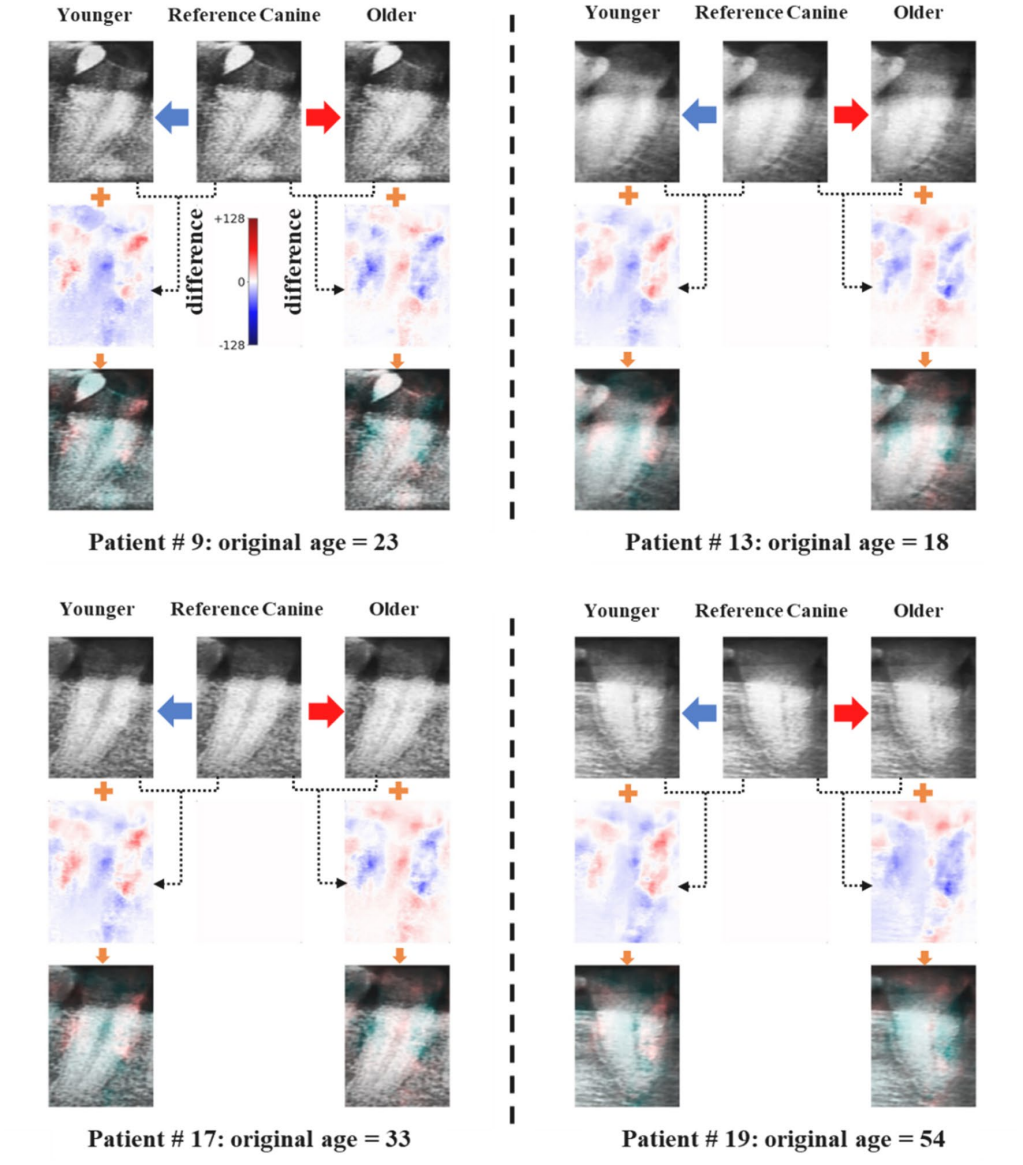
An example of color maps representing the correlation between advancing age and morphological changes in teeth, which were prepared by generating virtual canine teeth images when the subject was younger or older using the developed VAE and linear regression model.

The VAE decoder model is characterized by its ability to generate virtual images from latent variables that represent the characteristics of the target teeth. The coefficients of the regression model trained with both latent variables and chronological age data contained information regarding the correlation between age and the corresponding feature values. Accordingly, it is possible to selectively change only the latent variables that contain age information by adding or subtracting the regression coefficient to or from the latent variables extracted from the reference tooth image. The corrected variables are then reconstructed using the decoder; as a result, the correlation between the morphology of teeth and age can be visualized, as shown in Figs. 5 and 6.

Virtual dental images that reflected an increase or decrease in the age of the subjects were generated and analyzed based on the latent variables obtained from the first molar teeth, as shown in Fig. 5. Among the images in the first row, the second is a reference tooth image that was reconstructed from the original image. The first and third images were of the same teeth when the subjects were younger and older than the reference image. The images on the left were generated by subtracting the regression coefficient from the latent variables obtained from the reference teeth image and reconstructing them using a decoder. By contrast, the images on the right were generated by adding the regression coefficient to the latent variables obtained from the reference teeth image and reconstructing them using the decoder.

The images in the second row were generated by subtracting the pixel values of the reference image from those of the virtually generated images to determine how the morphology of teeth changed concerning the regression coefficient. Red refers to the case where the pixels are brighter in the virtually generated image than in the original image. Blue indicates where the pixels are darker in the virtually generated image than in the original image. In addition, when the intensity of the color was higher, there was a larger difference in the pixels in the corresponding region. The images in the third row were generated by overlaying the obtained color maps onto the corresponding tooth images to analyze the morphological changes of the teeth by region. The intensity of the color in this color map indicates the amount of variation in the image pixels based on the regression coefficient. Thus, regions with higher color intensity were considered to affect the age estimation model outcomes significantly.

The values of the latent variables that were highly correlated with age were selectively increased by adding a regression coefficient to the latent variables extracted from the reference image. Corrected variables were reconstructed using a decoder. The results showed that the pulp cavity of the first molar teeth appeared brighter, and the dental cervical region became darker with age. These analysis results based on virtually generated tooth images were consistent with clinical results demonstrating that the pulp cavity became smaller with age advancement. Thus increasingly radiopaque, the cervical region became more radiolucent due to dental cervical abrasion and alveolar bone resorption^25,26^. The same analysis procedures were applied to the canine tooth images, and the results are shown in Fig. 6. For canine teeth, the pulp cavity appeared brighter, and the dental cervical region became darker as the subject grew older, similar to the results obtained from first molar teeth, even though morphological changes were not as pronounced as in the first molar teeth.

In this study, the difference in pixels between the virtually generated images and the reference image was estimated and then expressed as different colors in the form of a color map. Using this color map, a method for determining the correlation between each part of the teeth and the estimated age was proposed. The difference in pixels between images indicates which parts of the teeth affected the age estimation process and the significance of the effects. The method proposed in the present study was found to provide improved interpretability with higher resolution compared to the existing grad-CAM methods used to interpret CNN results. The developed method can also visualize the correlation between morphological changes in the teeth and advancing age.

## Discussion

Several studies have been conducted to find methods to estimate the age of subjects using dental radiographs and deep learning algorithms, as well as to determine which part of the teeth was more significantly affected by age, which is important in age prediction. Most previous studies have employed CNN-based models to estimate the age of subjects using dental radiographs and attempted to visualize the parts of teeth that affect the age estimation process via various techniques, such as Grad-CAM methods. This study investigated the use of a model that combined a VAE, which is well known for its good performance in image generation, and linear regression to more accurately represent the correlation between human aging and changes in the morphological features of teeth. In addition, the present study developed a parallel VAE with a linear regression model and verified that the model had a better age-estimation performance.

Two types of age prediction models, which combined VAE with linear regression models, were developed to determine the effect of learning methods on age prediction performance. For the single VAE-based model, a single VAE was trained using a combined dataset of mixed first molar and canine tooth images. In contrast, a parallel VAE-based model is composed of two VAEs to extract the unique and common features of the two types of teeth in parallel.

The subjects' ages were estimated based on their first molar and canine tooth images using the two estimation models developed in the present study. The median absolute error (MAE) of the single VAE-based model was 9.5, which was higher than that of the parallel VAE-based model at 6.99, indicating that the parallel VAE model was more accurate. This confirmed that the parallel VAE-based model, in which two types of images were learned in parallel, was more effective for age estimation than the single VAE-based model. The parallel VAE-based model had a better age-estimation performance because the latent variables extracted for the parallel VAE and used in the regression more accurately captured the correlation between the variables and age, compared to those of the single VAE. This finding aligned with the results of previous studies that the common latent space structure of a parallel VAE is effective in disentangling specific latent variables such as age from other variables when given the input data^22,27,28^.

The present study also proposed a method for visualizing the correlation between morphological changes in teeth that occur with advancing age by controlling the latent variables using regression coefficients. Specifically, when adding or subtracting the regression coefficient to or from the original latent variables generated from the reference image, the values of the latent variables that strongly correlate with age tend to change significantly. The morphological changes in the teeth with age can then be visualized by reconstructing the affected variables using a decoder. Subsequently, the differences in pixels between the virtually generated images and the original image are estimated and expressed as different colors in the form of a color map. The color maps indicate that the pulp cavity, cervical, and alveolar bone regions significantly affected age estimation.

Additional images were obtained by overlaying the obtained color maps onto the generated teeth images, which confirmed that the pulp cavity appeared brighter as the subject grew older, indicating that the two factors have a positive correlation. In contrast, the cervical region became darker as the subjects grew older, indicating a negative correlation. These analysis results were consistent with the clinical results. They demonstrated that the pulp cavity became smaller with advancing age due to pulp calcification, dental cervical abrasion increased, and loss of alveolar bone occurred^25,26^. The chronological age estimation model using dental radiographs of the first molar and canine teeth provided excellent prediction accuracy; however, further research to improve the estimation accuracy, especially for children and elderly subjects, is required for this model to be applied to clinical applications in practice. In addition, further research needs to be performed in future work to dynamically optimize the hyperparameters of the model to optimize the weight values for the parallel VAE loss function.

In this study, methods were proposed for identifying the parts of teeth that more significantly affect the age estimation process and quantitatively analyzing the morphological changes in teeth with age. This was accomplished using color maps representing pixel differences between virtual images of teeth, generated by increasing or decreasing the age of the subjects. These methods of controlling latent variables using regression coefficients and visualizing the resultant changes are expected to be employed as a new approach to determine and analyze in detail regions of interest in images that are closely related to specific variables, particularly in medical deep learning. In addition, since the amount of variation in a given image can be visualized for changes in certain variables using this approach, the significant findings of this study are expected to contribute to the development of explainable AI.

## Data Availability

The datasets used and analyzed during the study are available from the corresponding author upon reasonable request.

## References

[1] Kim S, Lee YH, Noh YK, Park FC, Auh Q (2021). Age-group determination of living individuals using first molar images based on artificial intelligence. Sci. Rep..

[2] Lee JH (2017). Morphological analysis of the lower second premolar for age estimation of Korean adults. Forensic Sci. Int..

[3] Zelic K, Pavlovic S, Mijucic J, Djuric M, Djonic D (2020). Applicability of pulp/tooth ratio method for age estimation. Forensic Sci. Med. Pathol..

[4] Dallora AL, Anderberg P, Kvist O, Mendes E, Diaz Ruiz S, Sanmartin Berglund J (2019). Bone age assessment with various machine learning techniques: A systematic literature review and meta-analysis. PLoS ONE.

[5] Vila-Blanco N, Carreira MJ, Varas-Quintana P, Balsa-Castro C, Tomas I (2020). Deep neural networks for chronological age estimation from OPG images. IEEE Trans. Med. Imaging..

[6] Han Y, Wang G (2020). Skeletal bone age prediction based on a deep residual network with spatial transformer. Comput. Methods Programs Biomed..

[7] Kahaki SM, Nordin M, Ahmad NS, Arzoky M, Ismail W (2020). Deep convolutional neural network designed for age assessment based on orthopantomography data. Neural. Comput. Appl..

[8] Guo YC (2021). Accurate age classification using manual method and deep convolutional neural network based on orthopantomogram images. Int. J. Legal Med..

[9] Han M (2022). With or without human interference for precise age estimation based on machine learning?. Int. J. Legal Med..

[10] Spampinato C, Palazzo S, Giordano D, Aldinucci M, Leonardi R (2017). Deep learning for automated skeletal bone age assessment in X-ray images. Med. Image Anal..

[11] Yoon SJ, Hyong Kim T, Joo SB, Eel Oh S (2020). Automatic multi-class intertrochanteric femur fracture detection from CT images based on AO/OTA classification using faster R-CNN-BO method. J. Appl. Biomed..

[12] Alyafeai Z, Ghouti L (2020). A fully-automated deep learning pipeline for cervical cancer classification. Expert Syst. Appl..

[13] Milošević D, Vodanović M, Galić I, Subašić M (2022). Automated estimation of chronological age from panoramic dental X-ray images using deep learning. Expert Syst. Appl..

[14] Zhang, Z., Song, Y. & Qi, H. Age progression/regression by conditional adversarial autoencoder, in *Proceedings of the IEEE Computer Society Conference on Computer Vision and Pattern Recognition*, 5810–5818 (2017).

[15] Zhao Q, Adeli E, Honnorat N, Leng T, Pohl KM, Shen D, Liu T, Peters TM, Staib LH, Essert C, Zhou S, Yap P-T, Khan A (2019). Variational Autoencoder for Regression: Application to Brain Aging Analysis. Medical Image Computing and Computer Assisted Intervention.

[16] Kazmi S, Mânica S, Revie G, Shepherd S, Hector M (2019). Age estimation using canine pulp volumes in adults: a CBCT image analysis. Int. J. Legal Med..

[17] Tardivo D (2011). Three-dimensional modeling of the various volumes of canines to determine age and sex: a preliminary study. J. Forensic Sci..

[18] Marroquin TY (2017). Age estimation in adults by dental imaging assessment systematic review. Forensic Sci. Int..

[19] Ai B (2020). Convolutional neural network to retrieve water depth in marine shallow water area from remote sensing images. IEEE J. Sel. Top. Appl. Earth Obs. Remote Sens..

[20] Yan S, Smith JS, Lu W, Zhang B (2018). Abnormal event detection from videos using a two-stream recurrent variational autoencoder. IEEE Trans. Cognit. Dev. Syst..

[21] Soomer H, Ranta H, Lincoln MJ, Penttila A, Leibur E (2003). Reliability and validity of eight dental age estimation methods for adults. J. Forensic Sci..

[22] Hu D (2020). Disentangled-multimodal adversarial autoencoder: Application to infant age prediction with incomplete multimodal neuroimages. IEEE Trans. Med. Imaging.

[23] Nasser Y, Jennane R, Chetouani A, Lespessailles E, El Hassouni M (2020). Discriminative regularized auto-encoder for early detection of knee osteoarthritis: Data from the osteoarthritis initiative. IEEE Trans. Med. Imaging.

[24] Cunha E (2009). The problem of aging human remains and living individuals: a review. Forensic Sci. Int..

[25] Papapanou PN, Wennström JL, Gröndahl K (1988). Periodontal status in relation to age and tooth type: A cross-sectional radiographic study. J. Clin. Periodontol..

[26] Kvaal SI, Kolltveit KM, Thomsen IO, Solheim T (1995). Age estimation of adults from dental radiographs. Forensic Sci. Int..

[27] Cheng J, Gao M, Liu J, Yue H, Kuang H, Liu J, Wang J (2021). Multimodal disentangled variational autoencoder with game theoretic interpretability for glioma grading. IEEE J. Biomed. Health. Inf..

[28] Yue H, Liu J, Li J, Kuang H, Lang J, Cheng J, Peng L, Han Y, Bai H, Wang Y, Wang Q (2022). MLDRL: Multi-loss disentangled representation learning for predicting esophageal cancer response to neoadjuvant chemoradiotherapy using longitudinal CT images. Med. Image Anal..

